# The 21st-century adolescent mothers’ profile and adolescent pregnancy reduction in Brazil

**DOI:** 10.1590/1806-9282.20250517

**Published:** 2026-03-30

**Authors:** Denise Leite Maia Monteiro, Ida Peréa Monteiro, Zenilda Vieira Bruno, Roberta Monteiro Raupp, Fagner Ferreira Gonçalves, Gabriella de Oliveira Flor Ferreira, Letícia Freitas Simões, Aylana Ramos Gomes de Oliveira

**Affiliations:** 1 Universidade do Estado do Rio de Janeiro – Rio de Janeiro (RJ), Brazil.; 2 Centro Universitário Serra dos Órgãos – Teresópolis (RJ), Brazil.; 3 Associação Brasileira de Obstetrícia e Ginecologia da Infância e Adolescência (Sogia-BR), São Paulo (SP), Brazil.; 4 Centro Universitário São Lucas – Porto Velho (RO), Brazil.; 5 Department of Municipal Health – Porto Velho (RO), Brazil.; 6 Universidade Federal do Ceará, Faculty of Medicine – Fortaleza (CE), Brazil.; 7 Instituto de Comunicação e Informação Científica e Tecnológica em Saúde, Oswaldo Cruz Foundation – Rio de Janeiro (RJ), Brazil.; 8 Centro Universitário Celso Lisboa – Rio de Janeiro (RJ), Brazil.

**Keywords:** Adolescent pregnancy, Sociodemographic factors, Fertility, Youth, Brazil

## Abstract

**OBJECTIVE:**

The aim of this study was to assess the changes in the sociodemographic profile and maternal healthcare associated with adolescent deliveries in Brazil in 2000, 2011, and 2022.

**METHOD:**

Cross-sectional study by search on the Live Births Data System. Analyzed variables from mothers aged 10–19 years in Brazil: age, education, ethnicity, marital status, prenatal consultations, mode of delivery, and the country’s region.

**RESULTS:**

In 2000, there were 750,537 adolescents’ live births to adolescents in Brazil, and in 2022, there were 315,606 (58% reduction). There was a decrease in the number of married adolescent mothers, from 48 to 29.7% in 2011 and 25.5% in 2022. Regarding ethnicity, 231,369 (75%) pregnant adolescents in 2022 were Black and Brown, compared with 47.4% in 2000; the Indigenous adolescent deliveries have tripled (from 0.8 to 2.4%), with 7,526 births in 2022. Education improved substantially (30.3% in 2000 to 53.0% in 2011 and 80.6% in 2022) with at least 8 years of study. Adolescents with no prenatal care decreased from 6% in 2000 to 2% in 2022. There was an increase in cesarean births from 26.4 to 41.5%. Regionally, the age-specific fertility rate from 15 to 19 years in 2000 was higher in the North (111.7/1,000) and lower in the Southeast (71.4/1,000), in 2022, there was a decrease to 61.7/1,000 and 29.1/1,000, respectively.

**CONCLUSION:**

There was a decrease in the number of married adolescent mothers and an increase in the education level of adolescent mothers. However, the rise in cesarean births and racial disparities, with Black and Brown adolescents comprising ¾ of pregnancies in 2022, highlights ongoing challenges for health policies.

## INTRODUCTION

Adolescence is a period of changes, marked by sexual maturation and influenced by environmental, cultural, and socio-economic characteristics. Therefore, adolescent pregnancy has implications, especially for those living in unfavorable social contexts^1^; it is a phenomenon of great relevance for public health in Brazil, reflecting regional, racial, and socioeconomic inequities^2^.

It is undeniable that access to sexual and reproductive health care, comprehensive sexual education, and effective contraceptive methods has a fundamental role in early pregnancy prevention^2^.

Despite the advances in health and education policies, Brazil still has high rates of births among adolescents, reflecting regional, socioeconomic, racial, and educational inequities. Since 2001, a tendency of decrease can be observed; however, the numbers are still high, indicating the need for a better understanding of the factors that influence this reality. In 2000, the age-specific fertility rate (ASFR) from 15 to 19 years was 80.9 per 1,000 (80.9‰) adolescents. Data from 2022 indicate a rate of 48‰ adolescents, with a 40% reduction^3^.

The aim of this study is to analyze the changes in the sociodemographic profile and in maternal care associated with births from adolescent mothers in Brazil from 2000 to 2022, based on data from reference years (2000, 2011, and 2022). By identifying these changes, this study seeks to contribute to the development of more effective strategies of prevention and care, promoting equity and improvement for the health of young mothers.

## METHODS

This is a cross-sectional study based on live births (LBs) in Brazil in the years 2000, 2011, and 2022. The assessment included all LB declarations (LBD) referring to adolescent births in these two years. The LBDs are found on the databases of the Live Births Data System (SINASC)/Information Technology Department/Unified Health System (DATASUS) of Brazil’s Ministry of Health^4^.

The choice to analyze the year 2000 is for it being the last year of the twentieth century and for presenting the highest rate of births in adolescence^3^. Then, the data was compared with 2022, which represents the country’s full and revised data, when information was extracted from SINASC^4^. We also analyzed the year 2011 because it constitutes the middle of the studied period.

The variables analyzed were self-referred ethnicity, education, number of prenatal consultations, mode of delivery, marital status, and region of residence. The choice of variables is justified by the relevance of these indicators in the sociodemographic characterization, as they reflect inequalities in the access to services as well as cultural and social patterns related to the formation of early family units. The analysis of these variables, therefore, allows for robust statistical analysis aimed at identifying changes in the profile of adolescent mothers over time and their possible relation to the reduction of pregnancy incidence in this age group.

The Statistical Package for the Social Sciences (SPSS®) program, version 22.0, for Windows® was used in this study. Descriptive analysis tables were created and exported to Microsoft Excel 365, version 2402.

The study comprised female adolescents aged 10–19 years who had LB in Brazil in the years 2000, 2011, and 2022. The calculation of age-specific fertility rates (ASFRs) was based on Brazil’s population prospects, per gender and age, for the period 2000–2022 (Brazilian Institute of Geography and Statistics, IBGE)^5^. For race identification, the study utilized the concept used by IBGE of Black person, defined in the Racial Equality Statute as individuals who are self-declared Black or Brown or who adopt an analogous self-definition^6^.

Descriptive statistics were applied to summarize sociodemographic and obstetric characteristics for each reference year. ASFR for the 10–14 and 15–19 age groups were calculated using the number of live births in each age range divided by the estimated female population in the same age group and multiplied by 1,000.

The data used in the research are available in the DATASUS/SINASC database. As it is a public domain database, the submission of the project to the institution’s Research Ethics Committee is not required^4^.

## RESULTS

Table 1 shows a significant decrease in the rate of LB to adolescent mothers in Brazil. In 2000, adolescent births represented 23.4% of total births, whereas in 2022, they decreased to 12.3%, that is, a reduction of 58%.

**Table 1:** Frequency of age-specific fertility rate decrease among adolescents aged 10–14 and 15–19 years (2000 and 2022).

	Adolescents aged 10–14 years					
	2000			2022		
Country / Region	LB from mothers aged 10–14 years	Estimated population aged 10–14 years	ASFR/1,000	LB from mothers aged 10–14 years	Estimated population aged 10–14 years	ASFR/1,000
Brazil	28,973	8,614,988	3.4	14,293	7,487,417	1.9
North	4,209	774,499	5.4	3,412	833,594	4.1
Northeast	10,247	2,767,482	3.7	5,513	2,297,600	2.4
Southeast	8,375	3,319,266	2.5	3,174	2,814,528	1.1
South	3,672	1,170,974	3.1	1,015	954,868	1.1
Center-West	2,470	582,767	4.2	1,179	586,827	2
	**Adolescents aged 15–19 years**					
	**2000**			**2022**		
**Country/Region**	**LB from mothers aged 15–19 years**	**Estimated population aged 15–19 years**	**ASFR/1,000**	**LB from mothers aged 15–19 years**	**Estimated population aged 15–19 years**	**ASFR/1,000**
Brazil	721,564	8,920,682	80.9	301,313	8,041,597	37.5
North	84,552	756,861	111.7	53,577	867,894	61.7
Northeast	231,791	2,765,364	83.8	100,018	2,421,243	41.3
Southeast	255,001	3,569,784	71.4	89,363	3,074,194	29.1
South	92,021	1,209,427	76.1	32,030	1,037,548	30.9
Center-West	58,199	619,246	94.0	26,325	640,718	41.1

Source: IBGE; DATASUS/SINASC. LB: live birth; ASFR: age-specific fertility rate.

The SINASC database began in 1994, when the frequency of births among adolescents was 19.8% of total births. This number increased year after year until it reached 750,537 births in 2000, which represented 23.4% of the births that occurred in Brazil that year. In 2001, it dropped to 724,886 of the total 3,115,474 births (23.3%) and gradually decreased until 2010 (19.3%). It remained stable from 2010 to 2013 and decreased again in 2014 to 18.9%, and since then it has been decreasing each year, reaching 12.3% in 2022. The number of pregnant adolescents in the age group 10–14 years only started to decrease significantly in 2015, when 26,700 infants were born, and in 2022 it decreased to 14,293 (data not shown).

In absolute numbers, in 2000, there were 750,537 adolescent LB, with 28,973 LB in the age group 10–14 years (ASFR 3.4‰) and 721,564 LB in the age group 15–19 (ASFR 80.9‰). In 2011, there was a decrease to 560,888 births to adolescents, with 27,785 LB in the age group 10–14 years (ASFR 3.3‰) and 533,103 LB in the age group 15–19 years (ASFR 48.9‰). In 2022, there was a decrease to 1.9‰ and 37.5‰, respectively. Thus, there was a 50.7% reduction in the age group 10–14 years and a 58.2% reduction in the age group 15–19 years between 2000 and 2022.

In the North Region, the ASFR reduction in the age group 10–14 years was of 24.1 and 44.8% in the age group 15–19 years. The reduction in other regions is described in Table 2.

**Table 2:** Distribution of ASFR per 1,000 adolescents, per Regions of Brazil (2000–2022).

Country/region	Adolescents aged 10–14 years			Adolescents aged 15–19 years		
	2000	2022	Evolution % 2000–2022	2000	2022	Evolution % 2000–2022
Brazil	3.4	1.9	-44.1	80.9	37.5	-53.7
North	5.4	4.1	-24.1	11.7	61.7	-44.8
Northeast	3.7	2.4	-35.1	83.8	41.3	-50.7
Southeast	2.5	1.1	-56.0	71.4	29.1	-59.2
South	3.1	1.1	-64.5	76.1	30.9	-59.4
Center-West	4.2	2.0	-52.4	94.0	41.1	-56.3

Source: IBGE; DATASUS/SINASC.

The analysis of Table 3 shows that in 2000, there were 349,401 (48%) adolescent mothers who were married, in a consensual union, separated, or widowed, with a decrease to 29.7% in 2011 and to 25.5% in 2022.

**Table 3:** Socio-demographic characteristics of pregnant adolescents aged 10–19 years compared in 2000 and 2022.

Maternal variable	2000		2011		2022	
	Number	%	Number	%	Number	%
Marital status						
Single	377,486	52	388,723	70.3	232,868	74.5
Lives or has lived maritally	349,401	48	163,914	29.7	79,746	25.5
Ethnicity/race						
White	340,070	51	185,982	34.3	68,937	22.3
Brown and Black	315,692	47.4	349,414	64.5	231,369	74.9
Asian	5,180	0.8	1,121	0.2	1.21	0.4
Indigenous	5,255	0.8	5,567	1.0	7,526	2.4
Education						
None	24,429	3.4	2,598	0.5	1,203	0.4
1–7 years	471,213	66.3	243,792	44.3	59,282	19
8 and more	215,617	30.3	303,792	53.0	251,595	80.6
Prenatal consultation						
None	43,227	0.0	17,121	3.1	6,117	2.0
1–3 consultations	106,304	14.8	61,809	11.1	26,732	8.5
4–6 consultations	296,421	41.2	200,663	36.1	80,011	25.5
7 or more consultations	272,612	38	276,166	49.7	200,804	64
Mode of delivery						
Vaginal	549,326	73.6	340,907	61.0	184,531	58.5
Cesarean	197,211	26.4	218,452	39.0	130,872	41.5

Source: DATASUS/SINASC.

Regarding education, in 2000, there were 24,429 (3.4%) adolescents who referred having no education, 471,213 (66.3%) had 1–7 years of education, and 215,617 (30.3%) had ≥8 years of education. In 2011, these numbers changed to 53.9% having ≥8 years of education. In 2022, 251,595 (80.6%) had ≥8 years of education.

Regarding ethnicity, in 2000 there were 340,070 (51%) self-declared White, 315,692 (47.4%) Black and Brown, 5,180 (0.8%) Asian, and 5,255 (0.8%) Indigenous. In 2011, the rate of Black and Brown adolescent mothers increased to 64.5%, and in 2022, to 74.9%^4^. There is a prevalence in four of the country’s regions, with 85.7% in the North Region, 86.6% in the Northeast, 65.8% in the Southeast, 74% in the CenterWest; in the South, there were only 29.9%.

Regarding prenatal consultations, in 2000, there were 6% of pregnant adolescents with no consultation, 41.2% with 4–6 consultations and 38% with 7 or more consultations. In 2011, 3.1% had no consultation, and in 2022, 2% of pregnant adolescents did not receive prenatal care, indicating an improvement in prenatal care in adolescence.

Regarding the mode of delivery, in 2000, vaginal births were 549,326 (73.6%), in 2011, they were 340,907 (61.0%) and in 2022, there were 184,531 (58.5%). These data highlight an increase in the adolescent cesarean birth rate (Tables 3 and 4).

**Table 4:** Summary of the main changes in the profile of Brazilian pregnant adolescents (2000–2022).

Maternal variable	2000		2022	
	Number	%	Number	%
Marital status				
Lives or has lived maritally	349,401	48	79,746	25.5
Ethnicity/race				
Black and Brown	315,692	47.4	231,369	74.9
Education				
8 and more	215,617	30.3	251,595	80.6
Prenatal consultation				
None	43,227	6	6,117	2
7 or more consultations	272,612	38	200,804	64
Mode of delivery				
Vaginal	549,326	73.6	184,531	58.5

Source: DATASUS/SINASC, 2025.

## DISCUSSION

This is the first study assessing the change in the profile of Brazilian pregnant adolescents in the 21st century. Adolescent pregnancy reduction started in 2001, when there were 724,886 adolescent LB^4^, marking the beginning of a downward trend that continued throughout the study period. These data suggest that policies or social changes implemented around that time may have contributed to this reduction.

In Brazil, there was a 58% reduction of adolescent LB between 2000 and 2022 in all regions. The reduction in births in the group of mothers aged 10–14 only began to decrease from 2015 onward, when 26,700 infants were born, and in 2022 it decreased to 14,293^4,7^.

The decline observed in Brazil, particularly after 2014, reflects the global trend. For example, countries in sub-Saharan Africa have also reported declines in adolescent birth rates, although it remains the continent with the highest frequency of adolescent births. Compared to Brazil, where the decline was more pronounced in some regions, the African context presents different socioeconomic, cultural, and political challenges^8,9^.

In contrast, countries in Latin America and Southeast Asia demonstrated patterns more similar to Brazil, suggesting that region-specific policies and socio-economic development may play key roles. These comparisons highlight the relative success of Brazil’s recent actions and suggest that continued investment in youth-oriented health policies may further accelerate this progress. According to the World Bank, in 2024, worldwide, pregnancy in adolescents aged 15–19 years has been decreasing over the years. In 2000, the world rate was 64.4‰, in 2022,41.8‰, and in 2024, 40.7‰. In Brazil, the analysis of this age group revealed that in 2022, the adolescent LB rate was 43.6‰. In South America, the highest rate corresponded to Venezuela with 82‰, and the lowest to Chile with 22.8‰^10^. In Brazil, despite the decrease, the numbers remain high.

Pregnancy in adolescents under 14 years of age is closely linked to sexual violence. It is noteworthy that sexual violence has a central role and causes severe psychological consequences, including depression, anxiety, and lifelong sexual disorders. In Brazil, the situation is comparable to countries such as South Africa, where sexual violence is strongly related to unplanned pregnancies among girls and young women^8,11^.

Socio-demographic analyses of adolescent mothers in Brazil reveal a significant change in marital status in the past decades. In 2000, half of these young women were married or lived maritally, and in 2022, this figure dropped to 25.5%, reflecting a continued decline in early unions. While direct comparisons with other countries must be made with caution due to differing age groups and definitions, these figures contrast with global patterns where early marriage and adolescent pregnancy remain prevalent. For instance, in Mozambique, 48% of women aged 20–24 were married before the age of 18, and 40% became pregnant during adolescence. Although the indicators differ, both highlight the persistent challenges related to early reproductive health outcomes in diverse socio-cultural contexts^9^.

Brazil advanced in the protection of children’s and adolescents’ rights when promulgating Law 13,811/2019, which forbids marriage under age 16 years^12^. An analysis of this issue in the period 2018–2022 highlighted a 30.5% reduction of underage marriages, from 17,753 in 2018 to 12,509 in 2022^13^. Child marriage is any formal or informal union before the age of 18 years^14^.

In Brazil, the improvement of adolescent mothers’ education is a positive indicator, with a significant reduction in the proportion of young mothers with no education and an increase in those with at least 8 years of education, which favors labor market insertion and better living conditions^15^. Analysis of SINASC data shows a true upward shift in schooling levels within each age group. Among girls aged 10–14 years, the proportion with 8–11 years of schooling rose from 2.4% in 2000 to 20.8% in 2011 and 48.7% in 2022, while illiteracy declined from 4.3 to 0.9%. A similar trend occurred among adolescents aged 15–19 years: those with 8–11 years of schooling increased from 25.6% in 2000 to 79.6% in 2022, and illiteracy decreased from 3.2 to 0.3%^4^.

The increase in the educational level of adolescent mothers observed in recent years may reflect the positive effects of broader structural interventions, such as social protection programs that have increased school attendance and retention, especially among low-income and marginalized populations^15,16^.

The relation between education and adolescent pregnancy is corroborated by national and international studies. In Brazil, adolescent pregnancy is strongly associated with school dropout, often due to lack of family support and economic pressure, which reduces the chances of educational continuity for these young women^16^. Beyene et al. stress that the increase of education and access to job opportunities are factors of protection against early pregnancy and highlight the importance of education and reproductive health programs^17^.

Another relevant advancement is the increase in prenatal care, with the proportion of adolescents who had seven or more consultations increasing from 38 to 64% in 2022. This evolution suggests an improvement in health coverage, considering that early pregnancy is associated with risks, such as preterm labor and low birth weight^18^.

However, regarding the mode of delivery, in 2022 there was an increase in cesarean births. Although vaginal births continue to prevail, the increase of cesarean births among adolescents is a matter of concern. According to the National Agency of Supplementary Health, unnecessary indication of cesarean birth poses health risks to mother and child, increasing preterm labor, respiratory problems in the newborn infant, and death risk to the parturient^19^. The increase in cesarean births among adolescents may reflect a general upward trend in cesarean deliveries across all age groups in Brazil, rather than a specific shift in adolescent obstetric care.

Our results indicate in relation to ethnicity/race that adolescent mother rates were consistently higher among self-declared Black and Brown women across most regions of Brazil, with the exception of the South. This fact is justified by regional characteristics, such as the high number of Italian and German immigrant descent, which resulted in a mostly White population^4^.

On the other hand, Dumas et al. studied adolescents aged 15–17 years in Louisiana and verified equal birth rates regardless of race. This investigation raises important questions about the interaction between race, socio-economic status, and family status, and local culture^20^.

In Brazil, the 2022 Census of IBGE shows that 45.3% of the population was self-declared Brown, 10.2% Black, 43.5% White, 0.8% Indigenous, and 0.4% Asian. It was the first time, since 1991, that there was a prevalence of self-declared Black and Brown population, with 55.5% of the total, which has a clear impact on the rate increase of Black and Brown pregnant women^21^.

However, although this is a relevant reason to justify the increase, this population is also the most susceptible regarding sexual violence. According to the 17th Brazilian Yearbook of Public Security, in Brazil, one rape occurs every 6 min, with 83,988 rape victims registered in 2023, with a rate of 41.4/100,000. Of these, 61.6% were under 14 years old, 88.2% were female, 52.2% were Black and Brown, and 86.4% of the aggressors of victims up to 13 years old were family members or acquaintances of the victims^22^. This scenario reinforces the need of public health policies that address structural inequalities and the racial and ethnic nuances in the outcomes of maternal health^23^.

We highlight the following limitations of this study: (a) insufficient filling of official data available on SINASC/DATASUS, which may affect the veracity of the information; (b) the impossibility to obtain the actual number of adolescents who had abortions and to identify the total number of stillbirths in adolescents, which restricted the analysis to LB of adolescent mothers; (c) the study is cross-sectional, which prevents us from establishing causal relationships, such as determining what is the cause or consequence of early motherhood; and (d) the use of different data sources to estimate the resident population by age group (DATASUS and IBGE), which suggests that our data may be underestimated.

The data analyzed in this study reflect positive changes in adolescent LB rate, such as improvement in prenatal care and education.

## CONCLUSION

Brazil presents a decrease in the adolescent LB rate, as well as in the fertility rate in this group. The decrease in the number of early unions, the increase in years of education, and the improvement of prenatal care in the group of adolescent mothers demonstrate the influence of changes in the adolescent mothers’ profile in the 21st century regarding adolescent pregnancy reduction in Brazil. However, the frequency of adolescent pregnancy remains high, with regional, educational, and racial/ethnic inequalities, which must be tackled in order to ensure equity in health for all female adolescents.

## Data Availability

The datasets generated and/or analyzed during the current study are available from the corresponding author upon reasonable request.
